# African Nutmeg (*Monodora Myristica*) Lowers Cholesterol and Modulates Lipid Peroxidation in Experimentally Induced Hypercholesterolemic Male Wistar Rats

**Published:** 2015-06

**Authors:** Nwozo Sarah Onyenibe, Kasumu Titilayo Fowokemi, Oyinloye Babatunji Emmanuel

**Affiliations:** 1 Nutritional and Industrial Research Laboratories, Department of Biochemistry, Faculty of Basic Medical Sciences, College of Medicine, University of Ibadan, Ibadan 200002, Nigeria;; 2 Department of Biochemistry, College of Sciences, Afe Babalola University, PMB 5454, Ado-Ekiti 360001, Nigeria

**Keywords:** antioxidant, Cholesterol, hypercholesterolemia, hypolipidemic, Monodora myristica, rats

## Abstract

To evaluate the cholesterol lowering potential and protective ability of aqueous extract of *Monodora myristica* experimental hypercholesterolemic rats, a short-term study was conducted. Hypercholesterolemia was induced by administering cholesterol orally at a dose of 40 mg/kg/0.3 ml. Plant extracts 100 or 200 mg/kg body weight and Questran 0.26 g/kg were administered five times a week for eight weeks for amelioration. Hypolipidemic effects were evaluated by measuring total cholesterol (TC), low-density lipoprotein cholesterol (LDL-C), high-density lipoprotein cholesterol (HDL-C) and triglycerides (TG) in the serum, while the protective ability was measured by the extent of lipid peroxidation (LPO) as well as enzymatic and non-enzymatic antioxidants levels in post mitochondrial fractions (PMF) of the hepatic and cardiac homogenates. Serum aminotransferases activities were also monitored. Results obtained shows that treatment with *M. myristica* elicited a significant reduction in serum TC, TG and LDL-C levels while there was concomitant increase in HDL-C of hypercholesterolemic rats. Elevations in serum aminotransferases activities and LPO level were reversed and a significant amelioration was noticed in enzymatic and non-enzymatic antioxidants status in the liver and heart of hypercholesterolemic rats. This study suggests that *M. myristica* possess cholesterol lowering potentials and protective ability in experimental hypercholesterolemia rat model.

## INTRODUCTION

Hypercholesterolemia is a major health challenge and the continuous ingestion of high amounts of fat seems to be directly related to hyperlipidemia in humans. Animal models have been used to have a better understanding of the relationship between disorders in cholesterol metabolism and atherogenesis and to test possible treatments for the reduction of circulating cholesterol level (1).

*Monodora myristica* Dunal is a perennial edible plant of the family Annonaceae. It is found most commonly in the evergreen forests of West Africa and common names are African nutmeg, calabash nutmeg, and in Nigeria, it is called ehuru, ariwo, ehiri, airama (2). Its seed has an odour and taste that is similar to nutmeg and is used as a popular spice in the West African cuisine. *M. myristica* tree grows naturally in evergreen forests in countries like Liberia, Nigeria, Cameroon, Angola, Uganda and west Kenya. This tropical shrub is of the family of flowering plants (3). *M. myristica* fruit is a berry of 20 cm diameter; it is smooth, green and spherical, and becomes woody at maturity. It is attached to a long stalk which is up to 60 cm long. Inside the fruit are the numerous oblong, pale brown seeds which are usually 1.5 cm long and are surrounded by a whitish fragrant pulp.

Studies have shown that almost every part of *M. myristica* tree is important economically. The timber is hard, easy to work with and is used for carpentry, house fittings and joinery while the seeds are also made into necklaces (5). The most economically important parts are the seeds which are embedded in a white sweet smelling pulp of the sub-spherical fruit. It has been observed that an average of 119-122 seeds can be found in one fruit (2). After harvesting, various unit operations such as fermentation, washing, drying and cracking are performed before consumption or storage.

The essential oil from the leaves contains *β*-caryophyllene, *α*-humulene and *α*-pinene, while that from the seeds contains *α*-phellandrene, *α*-pinene, myrcene, limonene and pinene (5). Phytochemical screening carried out on *M. myristica* extract revealed the presence of Tannin, saponin, flavonoid, steroid, terpenoids, cardiac glycoside, alkaloid and phenol. Earlier determination of the chemical constituents of the seeds revealed the presence of Fiberro-latic oils, resins, terpene, lactose, arocine, saponins, flavonoids and tannins (6).

Reports abound in the literature as to the medicinal use of *M. myristica*, the stem bark is used in the treatments of hemorrhoids, stomach ache, fever pains and eye diseases (7), while the seeds are used in treating headache and hypertension in Central African Republic (8). In Eastern Nigeria, the seeds are used as condiment and one of the spices used as postpartum tonic. *M. myristica* has been proven to have anti-sickling properties (7). When grounded to powder, the kernel is used to prepare soup as stimulant to relieve constipation and control passive uterine haemorrhage in women immediately after child birth (9, 10). This berry also has diuretic properties and used for mild fever and antiseptic (10). These considerations have prompted us to investigate the effect of this seed extract on experimental hypercholesterolemia and the accompanying oxidative stress in rats.

## MATERIALS AND METHODS

### Chemicals

Assay kits for cholesterol and high density lipoprotein cholesterol (HDL-c), triglycerides, alanine and amino transferases were obtained from Randox Laboratories Ltd, Ardmore, Co. Antrim, UK. Adrenaline, Thiobarbituric acid (TBA), Ellmans reagent (DTNB), Glutathione (GSH) and bovine serum albumin (BSA) were purchased from Sigma Chemical (St Louis, MO, USA). Dietary cholesterol was procured from a local vendor. Questran (Bristol-Myers Squibb, Hounslow, UK) was obtained locally from a Chemist in Ibadan, Nigeria. Other reagents used were of purest quality available.

### Plant material

Dried fruits of *Monodora myristica* were purchased locally from Bodija market, Ibadan, Nigeria and were identified at the Herbarium of Botany Department, University of Ibadan, Nigeria. It was powdered using hammer mill and was extracted by maceration in hot distilled water 72 h. Extract was filtered and concentrated on a Rotary evaporator to give dark brown concentrate which was used at concentrations of 100 and 200 mg/kg body weight.

### Animals

Thirty six male albino rats (Wistar strain) weighing between 120 g and 140 g were obtained from Primate Colony, Biochemistry Department and were housed in the Animal house, Biochemistry Department, University of Ibadan, Ibadan at normal room temperature. The rats were acclimatized for two weeks on standard diet (palletized Guinea feed, purchased from Guinea Feed, Ibadan, Nigeria). The animals were allowed free access to food and water *ad libitum.* Rats were randomly placed into six groups of Group A: Normal control and received only corn oil. Group B served as positive control and received only Questran. Group C animals received Standard drug (Questran) plus Cholesterol; Group D: Cholesterol only while groups E and F are treatment groups on cholesterol and plant extract at 100 and 200 mg/kg body weight respectively. Corn oil was used as vehicle for the administration of extract, Questran and cholesterol. Dietary cholesterol and Questran were given at doses of 40mg/0.3ml/animal and 0.26 g/kg body weight, respectively (11), while aqueous extract of *Monodora myristica* was administered at a dose of 100 and 200 mg/kg body weight. All drugs were administered by oral gavage, five times a week for eight consecutive weeks.

### Sample Collection

The animals were fasted for 24 h after the last dose of extract and ethanol and were sacrificed by cervical dislocation. Blood was obtained using 2 ml syringe by cardiac puncture into clean bottles without anticoagulant and were left to stand for 1 h for complete coagulation. The clotted samples were spun at 3000 rpm for 10 minutes, the supernatant serum was removed and it was stored at 4°C. The visceral organs (liver and heart) were quickly removed, washed with 1.15 % KCl, homogenized in 56 mM Tris-HCl buffer (pH 7.4) containing 1.15% potassium chloride and the homogenate was centrifuged at 10,000 rpm for 15 minutes at 4°C. Supernatant was stored at 0°C until needed. Small pieces of liver and heart sections were fixed in 10 % formal saline and sent to Veterinary Anatomy Department, University of Ibadan, Ibadan for histopathological examination.

### Biochemical assays

Quantification of the protein was carried out using Biuret method (12) with bovine serum albumin (BSA) as standard. Lipid peroxidation was assayed by measuring thiobarbituric acid reactive substances (TBARS), by colorimetric reaction of the lipid peroxidation product malondialdehyde (MDA) with thiobarbituric acid (TBA) to form a pink precipitate, which was read at 532 nm by spectrophotometry. Catalase (CAT) activity was done by measuring the rate of decomposition of hydrogen peroxide at 570 nm as described by Sinha [1971] (13). Reduced glutathione (GSH) level was determined by measuring the rate of formation of chromphoric product in a reaction between DTNB (5,5´-dithiobis- (2-nitrbenzoic acid) and free sulphydryl groups at 412 nm (14). Superoxide dismutase (SOD) activity was assayed using the method of Misra and Fridovich (1972) (15), Cholesterol, HDL-c, triglyceride, AST and ALT was determined using Randox kit.

### Statistical analysis

All values were expressed as the mean ± S.D of six animals. Data were analyzed using one-way analysis of variance (Anova) followed by the post-hoc Duncan multiple test for analysis of biochemical data using SPSS (10.0) statistical software. *P* Values < 0.05 were considered statistically significant.

## RESULTS

### Effect of *Monodora myristica* on body weights of cholesterol fed rats

Table 1 shows data obtained for changes in body weight in experimental hypercholesterolemia rats model ameliorated with Questran (standard drug) and aqueous extracts of *M. myristica* (100 mg/kg b wt.). At the end of feeding experiment (8 weeks), group 1 (the normal control rats that were fed diet without cholesterol) showed 26.33% increase in body weight whereas group 4 (cholesterol only) rats showed 30.43% increase in body weight. Groups 2, 3, 5 and 6 (Questran or extract and cholesterol administered rats) showed less increase in body weight.

**Table 1:** Effect of *Monodora myristica* on body weights (g) of cholesterol fed rats

Groups	Body weight (g)		Weight gained (g) Percentage increase
	Initial weight	Final weight	

**Group 1 (Control)**	124.00 ± 7.58	156.66 ± 7.70	26.33
**Group 2 (Questran only)**	131.00 ± 6.53^a^ ^b^	159.00 ± 3.74	21.37
**Group 3 (Questran + cholesterol)**	126.00 ± 4.00	156.80 ± 6.00^b^	24.44
**Group 4 (Cholesterol only)**	138.00 ± 6.63^a^	180.00 ± 4.19^b^	30.43
**Group 5 (Cholesterol+ extract 100 mg)**	135.00 ± 4.00^b^	166.20 ± 4.00^b^	23.11
**Group 6 (Cholesterol+ extract 200 mg)**	140.00 ± 4.47^a^ ^b^	162. 00 ± 6.12^b^	15.71

Values are mean ± standard deviation, (where n=6);

a The mean is significant (*P*<0.05) when compared with control;

b The mean is significant (*P*<0.05) when compared with standard drug only.

### Effect of *Monodora myristica* on HDL-c, LDL-c, total cholesterol and triglyceride levels of cholesterol fed rats

Cholesterol administration caused a significant (*p*<0.05) elevation of serum total cholesterol and triglycerides in the animals as well as a slight increase in the level of LDL-c. Similarly a concomitant decrease in HDL-c was observed in group 4 (cholesterol only) as compared to the control. Administration of *M. myristica* (100 or 200 mg/kg bw) was able to reverse the obtained result (Table 2).

**Table 2:** Effect of *Monodora myristica* on HDL-c, LDL-c, total cholesterol and triglyceride levels of cholesterol fed rats

Groups	HDL-c (mg/dl)	LDL-c (mg/dl)	Total cholesterol (mg/dl)	Triglyceride (mg/dl)

**Group1 (Control)**	1.24 ± 0.61	0.95 ± 0.05	58.01 ± 5.71	38.41 ± 1.53
**Group 2 (Questran only)**	1.46 ± 0.09	1.14 ± 0.39	104.15 ± 5.27^a^ ^c^	44.66 ± 1.18^c^
**Group 3 (Questran + cholesterol)**	1.27 ± 0.43	1.01 ± 0.13	100.36 ± 1.96^a^ ^c^	43.48 ±1.55^c^
**Group 4 (Cholesterol only)**	0.25 ± 0.08	1.39 ± 0.14	132.41 ± 10.50^a^ ^b^	58.05 ± 0.59^a^ ^b^
**Group 5 (Cholesterol+ extract 100 mg)**	1.94 ± 0.07^a^ ^b^ ^c^	1.27 ± 0.25	99.38 ± 1.59^a^ ^c^	40.93 ± 0.84^c^
**Group 6 (Cholesterol+ extract 200 mg)**	1.96 ± 0.07^a^ ^b^ ^c^	1.08 ± 0.14	91.76 ± 0.85^a^ ^c^	35.89 ± 4.06^b^ ^c^

Values are mean ± standard deviation, (where n=6).

a The mean is significant (*P*<0.05) when compared with control,

b The mean is significant (*P*<0.05) when compared with standard drug only;

c The mean is significant (*P*<0.05) when compared with cholesterol.

### Effect of *Monodora myristica* on hepatic and cardiac SOD, CAT, GSH and MDA levels of cholesterol fed rats

Results presented in Table 3 and Table 4 shows that administration of cholesterol at a dose of 40 mg/kg/0.3 ml lead to a significant (*p*<0.05) elevation in the levels of both hepatic and cardiac LPO with a concomitant reduction in enzymatic and non-enzymatic (SOD, CAT and GSH) antioxidants status in the liver and heart of hypercholesterolemic rats. Treatment with standard drug (Questran) or *M. myristica* (100 or 200 mg/kg bw) showed a significant ameliorative effect (*p*<0.05) when compared with the control.

**Table 3:** Effect of *Monodora myristica* on hepatic SOD, CAT, GSH and MDA levels of cholesterol fed rats

Groups	SOD (μmol/min)	CAT (μmol/min)	GSH (μg/ml)	MDA (mg/dl)

**Group1 (Control)**	83.33 ± 4.01	275.67 ± 6.17	118.17 ± 1.01	2.87 ± 0.44
**Group 2 (Questran only)**	70.56 ± 1.32^a^	209.33 ± 7.68	117.83 ± 4.76	3.87 ± 0.49
**Group 3 (Questran + cholesterol)**	76.68 ± 0.46^a^ ^b^ ^c^	219.67 ± 3.18^a^	114.50 ± 2.78	3.87 ± 0.49
**Group 4 (Cholesterol only)**	68.04 ± 1.74^a^	207.33 ± 4.06^a^	88.333 ± 21.67	5.67 ± 0.73
**Group 5 (Cholesterol+ extract 100 mg)**	78.82 ± 1.50^b^ ^c^	274.67 ± 15.68^b^ ^c^	114.52 ± 2.78^c^	4.30 ± 3.79^a^ ^b^
**Group 6 (Cholesterol+ extract 200 mg)**	80.30 ± 1.52^b^ ^c^	277.78 ± 2.99^b^ ^c^	118.17 ± 1.01^c^	4.23 ± 1.15^a^ ^b^

Values are mean ± standard deviation, (where n=6).

a The mean is significant (*P*<0.05) when compared with control;

b The mean is significant (*P*<0.05) when compared with standard drug only;

c The mean is significant (*P*<0.05) when compared with cholesterol.

### Effect of *Monodora myristica* on tissue protein levels, AST and ALT activities in cholesterol fed rats

The effect of M. myristica on hepatic and cardiac protein levels as well as AST and ALT activities in cholesterol fed rats is presented in Table 5. Hypercholesterolemic rats showed an increased activity in serum aminotransferases (AST and ALT) activities. Following treatment with Questran or *M. myristica*, it was observed that the elevated levels of AST and ALT were significantly lowered (*P*<0.05) (Groups 2, 3, 5 and 6) when compared with the control rats (Group 1).

**Table 4:** Effect of *Monodora myristica* on cardiac SOD, CAT, GSH and MDA levels of cholesterol fed rats

Groups	SOD (μmol/min)	CAT (μmol/min)	GSH (μg/ml)	MDA (mg/dl)

**Group 1 (Control)**	85.86 ± 4.04	160.19 ± 3.55	120.67 ± 1.17	1.53 ± 0.23
**Group 2 (Questran only)**	78.93 ± 1.49	152.49 ± 4.29	117.33 ± 0.83	3.93 ± 0.15^a^ ^b^
**Group 3 (Questran + cholesterol)**	74.01 ± 1.96^a^	208.62 ± 4.68^a^	117.83 ± 0.85	4.33 ± 1.45^a^
**Group 4 (Cholesterol only)**	78.13 ± 4.00^a^	129.54 ± 74.82^a^	116.67 ± 0.44	6.33 ± 1.20^a^
**Group 5 (Cholesterol+ extract 100 mg)**	80.47 ± 1.35	212.92 ± 4.11^a^ ^b^	118.83 ± 2.85	3.37 ± 0.12^b^
**Group 6 (Cholesterol+ extract 200 mg)**	88.51 ± 1.32^b^	221.85 ± 5.03^a^ ^b^	121.33 ± 1.67	2.10 ± 0.38

Values are mean ± standard deviation, (where n=6).

a The mean is significant (*P*<0.05) when compared with control;

b The mean is significant (*P*<0.05) when compared with standard drug only;

**Table 5:** Effect of *Monodora myristica* on tissue protein levels, AST and ALT activities in cholesterol fed rats

Groups	AST (U/l)	ALT (U/l)	Liver protein conc. (mg/dl)	Heart protein conc. (mg/dl)

**Group 1 (Control)**	24.77 ± 0.62	7.90 ± 0.27	10.33 ± 0.60	30.01 ± 1.26^a^ ^b^ ^c^
**Group 2 (Questran only)**	15.83 ± 0.60^a^ ^b^ ^c^	6.13 ± 0.62^a^ ^b^ ^c^	13.00 ± 1.12	31.53 ± 0.98
**Group 3 (Questran + cholesterol)**	20.78 ± 1.12^c^	23.13 ± 0.75^a^ ^b^	16.73 ± 1.35^a^ ^b^	23.57 ± 0.97^a^ ^b^
**Group 4 (Cholesterol only)**	108.40 ± 2.14^a^ ^b^	29.20 ± 0.73^a^ ^c^	14.20 ±1.50^a^	24.83 ± 1.75^a^ ^b^
**Group 5 (Cholesterol+ extract 100 mg)**	92.07 ± 0.66^a^ ^b^ ^c^	6.35 ± 0.29^b^ ^c^	22.70 ± 1.30^a^ ^b^ ^c^	22.70 ± 1.30^b^
**Group 6 (Cholesterol+ extract 200 mg)**	89.35 ± 0.55^a^ ^b^ ^c^	6.35 ± 0.29^b^ ^c^	30.01 ± 1.26^a^ ^b^ ^c^	29.87 ± 1.66^c^

Values are mean ± standard deviation, (where n=6).

a The mean is significant (*P*<0.05) when compared with control;

b The mean is significant (*P*<0.05) when compared with standard drug only;

c The mean is significant (*P*<0.05) when compared with cholesterol.

### Liver tissue histopathology

**Figure 1:**
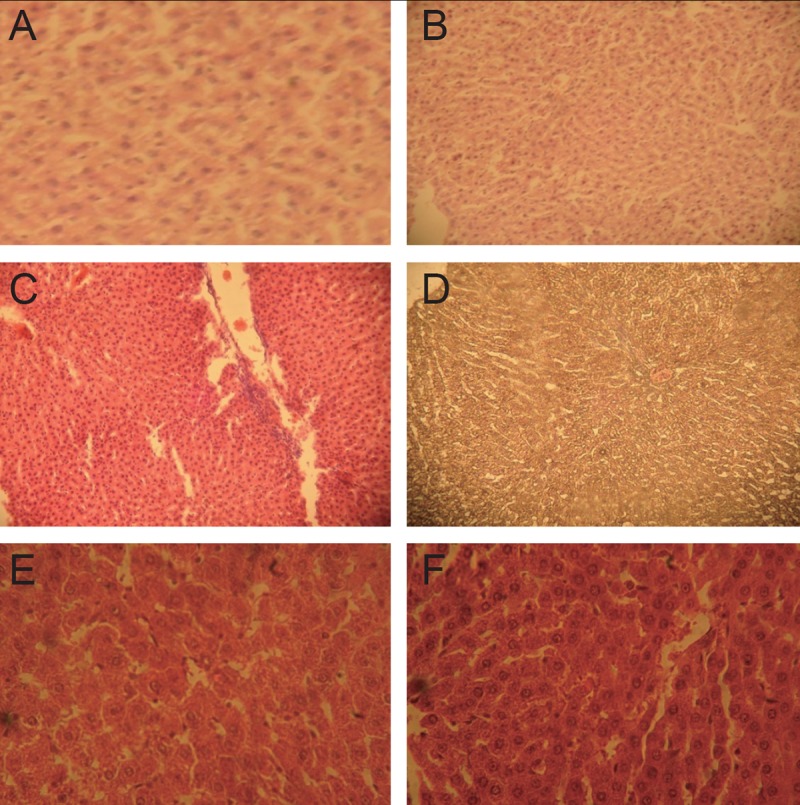
Histological analysis of liver sections. Liver tissues were stained with H&E (× 400). A, Control: showing normal liver histology, no abnormalities was seen; B, Rats receiving Questran (standard drug) showing prominent sinusoid; C, Rats receiving standard drug and cholesterol showing mild kupffer cell proliferation with moderate hepatic vacoular degeneration; D, Rats receiving cholesterol only showing marked portal congestion and vacuolar degeneration of the hepatocytes; E, Rats receiving cholesterol and *Monodora Myristica* at 100 mg/b. wt. showing moderate hepatic vacoular degeneration; F, Rats receiving cholesterol and *Monodora Myristica* extract at 200 mg/b. wt. showing moderate hepatic vacoular degeneration.

The results obtained from the histological studies of the liver tissue showing histopathological alterations are presented in Figure 1.

## DISCUSSION

The objective of the present study is to investigate the hypocholesterolemic potential and protective ability of *Monodora myristica* on cholesterol-induced hypercholesterolemic rats, using Questran as a standard hypolipidemic drug. Body weight changes in the animals were evaluated as a measure of growth performance and acceptability of treatment. The result obtained in this study suggests that the extract was able to control the increase in body weight. This was contrary to our previous reports where we had over 60% increase in body weight of rats administered cholesterol (11, 16). The observed reduction in body weight might be attributed to decrease in nutrient intake because of the high fat content of the cholesterol. Matos *et al*. and Hartvigsen *et al.* have proposed that high fat diet might impair the absorption of protein and other nutrients and this could culminate in decreased body weight (17, 18). Hypercholesterolemic animals co-treated with either the standard drug or plant extract had greater increase in body weight compared to negative control group. The improvement in body weight was however lower than those observed in animals on normal rat chow, thus implying the adverse effect of dietary cholesterol on their body weight.

Dietary lipids intakes have been known to alter lipid composition in the serum. Alterations in lipid profiles could provide information on the effect of the diet on lipid metabolism as well as predisposition to the development of atherosclerosis and other cardiovascular diseases. Elevated value of LDL-c has been pointed out as one of the risk factors for the development of atherosclerosis and related cardiovascular diseases (19). High serum triglycerides levels have also been reported to be an important risk factor as it influences lipid deposition clotting mechanism (20). LDL-c are the major transporters of cholesterol in the blood stream and are considered “bad cholesterol” because they carry fats out of the liver to the blood vessels and seem to encourage the deposition of cholesterol in the arteries. Similarly we observed a concomitant decrease in HDL-c in the group on cholesterol only. We obtained significant reduction in the levels of serum total cholesterol and triglycerides in hypercholesterolemic rats co-treated with *M. myristica* extract and this was lower than observed value for reference drug. Reduction in serum levels of total cholesterol could arise from impairment in β-oxidation of fatty acids or reduction in cholesterol absorption resulting from reduced incorporation into chyclomicrons, VLDL-c and LDL-c. HDL-c was significantly increased in hypercholesterolemic rats treated with plant extract. HDL-c is considered as good cholesterol as it is essential in the transportation of cholesterol to the liver for catabolism and a negative correlation between HDL-c and the risk of development of cardiovascular diseases. The significant decrease in total cholesterol and triacylglyceride as well as the slight decrease in LDL-c, which in essence increased high-density lipoprotein (HDL) cholesterol levels points to this plant as a potential hypolipidemic agent.

Reactive oxygen species are hydroxyl radical, superoxide anion radical, hydrogen peroxide and oxygen radical and they have been implicated in the pathogenesis of many diseases. The serum and tissues contains antioxidants that help combat oxidative stress. Increase in malondialdehyde (MDA) an index of lipid peroxidation in the liver and heart of cholesterol fed rats may be an indication of increased amount of oxidative stress in the cholesterol fed rats (Table 3 and Table 4). GSH plays an important role in the antioxidant effects, nutrient metabolism and regulation of cellular events (21). Cholesterol administration caused a significant decrease in GSH concentrations and the activities of SOD and CAT. The decrease in GSH reduces the antioxidative capacity and increase the capacity to respond to oxidative stress. GSH effectively scavenges free radicals and other oxygen species through nonenzymatic and enzymatic process. GSH and the dependent enzymes are one of the protective mechanisms against oxidative damage, both in blood and in the various tissues. There were significant increase in GSH, CAT and SOD by *Monodora myristica* extract in this study, the extract ameliorated oxidative stress induced by hypercholesterolemia due to its flavonoid and polyphenol content.

The liver is a major target organ for thyroid hormone with important biological and medical implications (22, 23). Protein metabolism has been known to be altered in diseased state. Protein concentrations in the hepatic and cardiac post mitochondrial fractions of hypercholesterolemic rats decreased, probably due to tissue damage and these were ameliorated by plant extract similar to normal control rats. Clinical diagnosis of disease and damage to the structural integrity of the liver is commonly assessed by monitoring the status of AST and ALT activities, which are sensitive serological marker enzymes of liver integrity (24). Higher activities of these enzymes in serum have been found in response to oxidative stress induced by high fat diets (22, 25).

The elevation of the AST and ALT level in cholesterol fed rats could be as a result of leakage of the enzymes into the serum as a result of tissue damage. Elevation in ALT and AST has been implicated as a risk factor in the development of cardiovascular disease and this was significantly reduced by the plant extract. These reports are consistent with histology result (Fig. 1) where we observed marked portal congestion and vacuolar degeneration of hepatocytes in the group treated with cholesterol only but in cholesterol co-administered standard drug treated group there was proliferation of kuffer cells and moderate hepatic vacuolar degeneration. Similar trends were observed in groups treated with the plant extract. This agrees with AST and ALT data which showed tissue injury in the rats fed with cholesterol and the elevated values were ameliorated by the plant extract.

In this study, cholesterol administration altered serum lipids, elicited oxidative stress and caused critical injury to the organ due to the overproduction of free radicals especially reactive oxygen species, which exert deleterious effects in organs. *Monodora myristica* produced favourable lipid profile, ameliorated antioxidant levels as well as serum AST and ALT activities. Histopathology results are in agreement with all the results discussed above, thus suggesting that aqueous extracts of *Monodora myristica* could reverse liver toxicity induced by high cholesterol diets and exert hypocholesteroleamic effect.

## References

[1] Moghadasian MH, Frohlich JJ, McManus BM (2001). Advances in experimental dyslipidemia and atherosclerosis. Lab Invest.

[2] Burabai W, Akor AJ, Igoni AH, Puyate YT (2007). Fracture resistance of African Nutmeg (*Monodora myristica*) compressive loading. Electron J. Environ Agric Food Chem.

[3] Okafor JC (1987). Development of forest tree crops for food supplies in Nigeria. Forest Ecol Management.

[4] Weiss EA (2002). Spice Crops.

[5] Nguefack J, Leth V, Amvam V, Zollo PH (2004). Evaluation of five essential oils from aromatic plants of Cameroon for controlling food spoilage and mycotoxin producing fungi. Int. J. Food Microbiol.

[6] Iwu MM (1993). Handbook of African medicinal plants.

[7] Uwakwe AA, Nwaoguikpe RN (2008). *In-vitro* antisickling effects of *Xylopia aethiopica* and *Monodora myristica*. Med Plant Res.

[8] Koudou J, Etou Ossibi AW, Aklikokou K, Abenna AA (2007). Chemical composition and hypotensive effects of the essential oil of *Monodora myristica* Gaertn. J. Biol. Sci.

[9] Udeala OK (2000). Preliminary evaluation of dike fat, a new tablet lubricant. J. Pharm Pharmacol.

[10] Iwu MM (2002). Evaluation of the anthihepatotoxic activity of the biflavonoids of *Garcina kola* Seeds. J. Ethnopharmacology.

[11] Nwozo SO, Orojobi F, Adaramoye OA (2011). Hypolipidemic and antioxidant potentials of *Xylopia aethiopica* seed extract in hypercholesterolaemic rats. J. Med Foods.

[12] Gornall AC, Bardawill EJ, David MM (1949). Determination of serum proteins by means of Biuret reaction. J. Biol. Chem.

[13] Sinha AK (1972). Colorimetric assay of catalase. Anal. Biochem.

[14] Beutler E (1963). Improved method of determination of blood glutathione. J. Lab Clin. Med.

[15] Misra HP, Fridovich I (1972). The role of superoxide anion in the autoxidation of epinephrine and a simple assay for superoxide dismutase. J. Biol Chem.

[16] Nwozo SO, Orojobi FB (2010). Hypolipidemic and antioxidant effects of *Tetrapleura tetraptera* fruits, including seeds in hypercholesterolaemic rats. Seed Sci. Biotechnol.

[17] Matos SL, Paula H, Pedrosa ML, Santos RC (2005). Dietary models for inducing hypercholesterolemia in rats. Brazilian Archives Biol Technol.

[18] Hartvigsen K, Binde CJ, Hansen LF, Rafia A (2007). A diet-induced hypercholesterolemic murine model to study atherogenesis without obesity and metabolic syndrome. Arterioscler Thromb Vasc Biol.

[19] Gertz GS, Reardon CA (2006). Diet and murine atherosclerosis. Arteriosclerosis, Thrombosis Vascular Biol.

[20] Harnafi H, Aziz M, Amrani S (2009). Sweet Basil (Ocimum basilicum L.) improves lipid metabolism in hypercholesterolemic rats. E Spen Eur E J. Clin. Nutr. Metab.

[21] Wu G, Fang YZ, Yang S, Lupton JR (2004). Glutathione metabolism and its implications for health. J. Nutr.

[22] Yang JJ, Li GL, Liu HR, Ren BB (2003). Effect of evening rose oil on activities of oxygen free radical scaverging-related enzymes and hepatic morphosis in rats on high lipid diet. J. NingXia Med Coll.

[23] Simon-Giavarotti KA, Giavarotti L, Gomes LF, Lima AF (2002). Enhancement of lindane-induced liver oxidative stress and hepatotoxicity by thyroid hormone is reduced by gadolinium chloride. Free Radic Res.

[24] Amin A, Hamza AA (2005). Oxidative stress mediates drug-induced hepatotoxicity in rats: A possible role of DNA fragmentation. Toxicology.

[25] Demori I, Voci A, Fugassa E, Burlando B (2006). Combined effects of high-fat diet and ethanol induce oxidative stress in rat liver. Alcohol.

